# Identification of dimethylamine monooxygenase in marine bacteria reveals a metabolic bottleneck in the methylated amine degradation pathway

**DOI:** 10.1038/ismej.2017.31

**Published:** 2017-03-17

**Authors:** Ian Lidbury, Michaela A Mausz, David J Scanlan, Yin Chen

**Affiliations:** 1 School of Life Sciences, Gibbet Hill Campus, University of Warwick, Coventry, UK

## Abstract

Methylated amines (MAs) are ubiquitous in the marine environment and their subsequent flux into the atmosphere can result in the formation of aerosols and ultimately cloud condensation nuclei. Therefore, these compounds have a potentially important role in climate regulation. Using *Ruegeria pomeroyi* as a model, we identified the genes encoding dimethylamine (DMA) monooxygenase (*dmmABC*) and demonstrate that this enzyme degrades DMA to monomethylamine (MMA). Although only *dmmABC* are required for enzyme activity in recombinant *Escherichia coli*, we found that an additional gene, *dmmD*, was required for the growth of *R. pomeroyi* on MAs. The *dmmDABC* genes are absent from the genomes of multiple marine bacteria, including all representatives of the cosmopolitan SAR11 clade. Consequently, the abundance of *dmmDABC* in marine metagenomes was substantially lower than the genes required for other metabolic steps of the MA degradation pathway. Thus, there is a genetic and potential metabolic bottleneck in the marine MA degradation pathway. Our data provide an explanation for the observation that DMA-derived secondary organic aerosols (SOAs) are among the most abundant SOAs detected in fine marine particles over the North and Tropical Atlantic Ocean.

## Introduction

Methylated amines (MAs) form part of the marine dissolved organic nitrogen pool and are ubiquitous in the marine environment. Their precursors, trimethylamine *N*-oxide (TMAO), glycine betaine, choline and carnitine are either osmolytes or constituents of lipid membranes within eukaryotic cells (Ikawa and Taylor, 1973; Treberg *et al.*, 2006). MAs (trimethylamine (TMA), dimethylamine (DMA) and monomethylamine (MMA)) form part of a trace gas mix that is constantly emitted from the oceans and collectively these trace gases have major implications for the climate, largely through the production of particulate marine aerosols (Carpenter *et al.*, 2012). Such aerosols can represent up to one-fifth of the total gaseous base compounds detected in the atmosphere over the oceans (Gibb *et al.*, 1999a). Their global annual flux is estimated to be ~80 Gg per year and their production in surface seawater, and subsequent emission into the atmosphere, is thought to be largely driven by biotic processes (Ge *et al.*, 2011). For example, over Cape Verde off the coast of West Africa, the accumulation of MAs in fine marine particles was positively correlated with algal blooms (Müller *et al.*, 2009). The flux of MAs into the atmosphere is important as they can undergo a number of different reactions resulting in a complex set of effects on the climate. For instance, they can influence the absorption and scattering of ultraviolet radiation, the formation of cloud condensation nuclei (Ge *et al.*, 2011), and the cloud droplet number concentration (Rinaldi *et al.*, 2010). Moreover, off the coast of California, during periods of elevated primary production, a shift in the composition of secondary organic aerosols (SOAs) toward amine-derived compounds resulted in an increase in cloud condensation nuclei activity (Sorooshian *et al.*, 2009). Thus, as a component of marine aerosols, MAs can actively affect the climate system.

**Figure 1:**
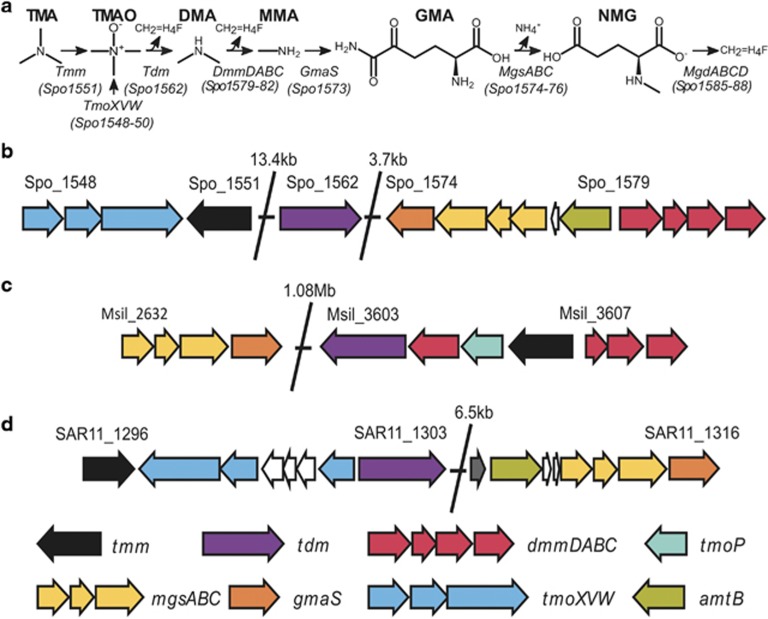
Scheme of (**a**) the proposed pathway of MA catabolism in *R. pomeroyi* DSS-3 and related MRC bacteria and (**b**) genomic regions encompassing the genes (*dmmDABC*) encoding the Dmm in *R. pomeroyi* DSS-3 and (**c**) *Methylocella silvestris* BL2. (**d**) *Candidatus* Pelagibacter ubique HTCC1062 does not possess *dmmDABC* in its genome despite containing all other genes required for TMA, TMAO and MMA degradation. *amtB*, ammonia transporter; CH_2_=H_4_F, 5,10-methylene tetrahydrofolate; DmmA, DmmB, DmmC, DmmD, DMA monooxygenase subunit A, B, C or D; GMA, gamma-glutamylmethylamide; GmaS, gamma-glutamylmethylamide synthetase; MgdABCD, *N*-methylglutamate dehydrogenase; MgsABC, *N*-methylglutamate synthase; NMG, *N*-methylglutamate; Tdm, trimethylamine *N*-oxide demethylase; Tmm, trimethylamine monooxygenase; *tmoP*, TMAO permease; TmoXVW, adenosine triphosphate-dependent TMAO transporter.

Historically, the *in situ* quantification of MAs in the marine environment has proven challenging. Consequently, there are only a few studies reporting their standing stock concentrations (Carpenter *et al.*, 2012). Generally, in surface seawater the concentration of MAs is in the nanomolar (nM) range, whereas in marine sediments it reaches low micromolar (μM) concentrations (Van Neste *et al.*, 1987; Gibb *et al.*, 1999b; Gibb and Hatton, 2004). Recent studies have identified a number of the key genes and enzymes catalyzing the degradation of TMA, TMAO and MMA in the marine environment (Chen *et al.*, 2010, 2011; Lidbury *et al.*, 2014) (Figure 1a). It is now known that bacteria capable of degrading MAs are abundant in surface seawater and are primarily related to the *Alphaproteobacteria* (Chen *et al.*, 2011; Sun *et al.*, 2011). Despite their low standing stock concentrations, expression of the key genes and enzymes catalyzing the degradation of MAs has been observed in surface seawater from various oceanic regions (Lidbury *et al.*, 2014). Indeed, marine *Alphaproteobacteria* often heavily transcribe the TMAO-specific transporter suggesting that demethylation of TMAO to DMA may be a major process in surface ocean waters (Sowell *et al.*, 2008; Ottesen *et al.*, 2011, 2013; Williams *et al.*, 2012; Gifford *et al.*, 2013).

The marine *Roseobacter* clade (MRC) and SAR11 clade are two monophyletic groups of *Alphaproteobacteria* that use differing ecological strategies for growth (Luo *et al.*, 2013). Both of these clades can catabolize MAs in order to generate reducing power, whereas the MRC can also utilize these compounds as a sole source of both carbon and nitrogen (Chen, 2012). *Ruegeria pomeroyi* DSS-3, a member of the MRC, has been used as a model organism to study the degradation of TMA, TMAO and MMA. However, how these marine bacteria degrade DMA remains unknown. In the methylotrophic soil bacterium *Methylocella silvestris* BL2, a three-gene cluster (*dmmABC*) is required for growth of this organism on DMA, as mutants lacking *dmm* genes ceased to grow on DMA as sole nitrogen source (Zhu *et al.*, 2014). In addition, in another methylotrophic soil bacterium *Paracoccus aminophilus* JCM 7686, mutants lacking a functional *dmmABC* or an additional gene (*dmmD*), could no longer utilize DMA as a sole carbon source (Dziewit *et al.*, 2015). Furthermore, a DMA monooxygenase (Dmm) has been purified from MA-grown *Aminobacter aminovorans* cells and shown to be a nicotinamide adenine dinucleotide phosphate-dependent enzyme that produces MMA and formaldehyde with DMA being the most active substrate (Alberta and Dawson, 1987). Dmm has a native molecular weight of ~210 kDa and comprises three subunits 42 000, 36 000 and 24 000 Da in size, each of which are essential for *in vitro* activity (Alberta and Dawson, 1987).

Here, we set out to determine the genes catalyzing DMA demethylation in marine bacteria using *R. pomeroyi* DSS-3 as the model organism. Dmm was heterologously expressed in *Escherichia coli* and the function of the predicted three-gene cluster, *dmmABC*, was confirmed for the first time by enzymatic, chemical and growth assays. We also demonstrate that, unlike the genes required for the catabolism of TMA, TMAO and MMA, the genes required for DMA catabolism are absent from key marine bacterial taxa and are subsequently depleted in metagenomes derived from oceanic surface waters.

## Materials and methods

### Bacterial cultivation

The strains used in this study are listed in Supplementary Table S1. *R. pomeroyi* wild-type and mutants were grown in a marine ammonium minimal salts medium (Thompson *et al.*, 1995) with slight modifications (Lidbury *et al.*, 2015) using 10 mM glucose as carbon source. TMA, TMAO, DMA and MMA (1 mM) were added as sole nitrogen source. To observe growth on different nitrogen sources, cultures (*n*=3) were set up in 125 ml serum vials containing 25 ml medium. Overnight starter cultures were harvested by centrifugation (1500 × *g*, 5 min) and washed three times in nitrogen-free marine ammonium minimal salts before inoculation (8% v/v). Cultures were kept under constant agitation (150 r.p.m.) at 30 °C.

### Overexpression of *dmmABC* and *dmmDABC* in a heterologous host

All primers used in this study are listed in Supplementary Table S2. Either *dmmABC* encoding the structural components of Dmm or the entire operon *dmmDABC* were subcloned into the pGEM-T EASY vector (Promega, Southampton, UK). Sequence integrity was checked before digestion using the restriction enzymes *Nhe*I and *Hin*dIII and subsequent ligation into the expression vector pET28a, which was transformed into *E. coli* BLR(DE3)pLysS (Promega). Transformed *E. coli* cells were grown for 32 h at 25 °C in the presence of 0.2 mM isopropyl β-D-1-thiogalactopyranoside and 1 mM DMA.

### Mutagenic analysis and mutant complementation in R. pomeroyi

A *dmmD* disrupted mutant (*dmmD::Gm*) in *R. pomeroyi* DSS-3 was constructed by cloning part of the gene (Spo1579) into the pGEM-T EASY vector. A gentamicin resistance cassette (Dennis and Zylstra, 1998) was inserted into a naturally occurring *Spe*I site located near the centre of the gene. The mutated construct was cloned into the suicide vector, pk18mobsacB (Schäfer *et al.*, 1994), and mobilized into *R. pomeroyi* via conjugation with *E. coli* S17-1 electrocompetent cells. Transconjugants were streaked onto gentamicin plates containing MMA as the sole nitrogen source to counterselect against *E. coli* (Lidbury *et al.*, 2014). Double homologous recombination events were selected for by transconjugant sensitivity to kanamycin. The mutation was confirmed by PCR and sequencing.

To complement the *dmmD::Gm* with *dmmDABC* plus its native promoter, the entire gene cluster was amplified introducing the restriction sites *Xba*I and *Kpn*I at the 5′ and 3′ ends, respectively. For complementation with the structural genes *dmmABC*, the promoter alone was amplified introducing the restriction sites *Xba*I and *Hin*dIII at the 5′ and 3′ ends, respectively. In addition, *dmmABC* was amplified introducing the restriction sites *Hin*dIII and *Kpn*I at the 5′ and 3′ ends, respectively. For complementation using just *dmmD*, this gene (Spo1579) plus the promoter were amplified introducing the restriction sites *Bam*HI and *Hin*dIII at the 5′ and 3′ end, respectively. All PCR fragments were subcloned into the pGEM-T EASY vector. Sequence integrity was checked before cloning the construct into the broad-host range plasmid pBBR1MCS-km (Kovach *et al.*, 1995) and mobilized into *dmmD::Gm* via conjugation as before. Transconjugants were selected by growth on half-strength Yeast Tryptone Sea Salts (1/2 YTSS) media containing 80 μg ml^−1^ kanamycin and 10 μg ml^−1^ gentamicin. Complementation was confirmed by PCR and sequencing.

### Quantification of MAs

Cells were boiled for ⩾10 min and debris was removed via centrifugation (17 000 × *g*, 5 min). TMA, TMAO, DMA and MMA were quantified on a cation-exchange ion chromatograph (881 Compact IC pro, Metrohm, Runcorn, UK) supplied with Metrosep C 4 guard (Metrohm, Switzerland) and Metrosep C 4-250/4.0 separation column, and a conductivity detector (Metrohm, Switzerland) using an external calibration (Lidbury *et al.*, 2014).

### Analysis of enzymes involved in MA metabolism in sequenced marine microbial genomes

Single amplified genomes used in this study derived from the Integrated Microbial Genome (IMG) database of the Joint Genome Institute (JGI) (https://img.jgi.doe.gov/cgi-bin/m/main.cgi). All available defined marine bacterial genomes were screened for enzymes catalyzing MA degradation using a BLASTP analysis with Tmm (Spo1551), Tdm (Spo1562), DmmD (Spo1579), DmmA (Spo1580), DmmB (Spo1581), DmmC (Spo1582), GmaS (Spo1573) and TmoX (Spo1548) from *R. pomeroyi* DSS-3 as query sequences using a stringent cutoff value of e-50. Marine bacterial genomes containing genes encoding these proteins are listed in Supplementary Table S3. Taxonomy information at the phylum, class and order level was exported from the IMG/JGI database. For phylogenetic analysis, amino-acid sequences of *dmmD*, *dmmA*, *dmmB* and *dmmC* from 36 taxa were aligned individually by MUSCLE (Edgar, 2004), trimmed at either end and combined to one alignment. Evolutionary analysis was conducted in MEGA7 (Kumar *et al.*, 2016) on a total of 1043 positions remaining in the data set after exclusion of gaps and missing data. A phylogenetic tree was inferred by a maximum likelihood approach applying the WAG model (Whelan and Goldman, 2001) with 999 bootstrap replicates and using a maximum parsimony tree derived from neighbor-joining as the initial tree.

### Analysis of enzymes involved in MA metabolism in marine metagenomes and metatranscriptomes

The metagenomes used in this study and the abundances of MA degradation genes are listed in Supplementary Table S4. Metagenomes were chosen from the IMG/JGI database and predominantly consisted of sites used in the global ocean sampling expedition (Rusch *et al.*, 2007). A BLASTP analysis was performed using a stringency of >30% identity and a cutoff value of e-50. Query sequences were identical to those described above. The number of retrieved sequences for each protein was normalized by dividing the length of the query by the length of RecA. Finally, the normalized hits were divided by the number of hits retrieved for two single copy genes (*recA* and *gyrB*) to obtain the percentage of MA-utilizing marine bacteria present at each site. For phylogenetic analysis, hits were clustered using CD-HIT (Huang *et al.*, 2010) at a similarity cutoff of 0.8. Representative sequences were then used as query in BLASTP (multiple query function) searches using the National Centre for Bioinformatics database (nr). The best hit was used to assign taxonomy at the family level.

The metatranscriptomes used in this study are listed in Supplementary Table S5. Metatranscriptomes deposited in the IMG/JGI database were used for a BLASTP analysis with a stringency level of >40% similarity and a cutoff value of e-20. Query sequences were identical to those used above and data normalized by the length of RecA as described above.

## Results

### Identification of a four-gene cluster in R. pomeroyi DSS-3

*R. pomeroyi* can utilize TMA, DMA and MMA as a sole nitrogen source (Lidbury *et al.*, 2015). Therefore, a BLASTP analysis on *R. pomeroyi* was performed to identify candidate genes involved in DMA catabolism using the three-gene cluster identified as *dmmABC* (Msil_3607, Msil_3608 and Msil_3609) from *M. silvestris* as the query sequences (Zhu *et al.*, 2014). Three open reading frames, Spo1580, Spo1581 and Spo1582 shared good homology with Msil_3607 (E-value, 4.0e-32; 38.92%), Msil_3608 (E-value, 4.0e-75; 41.07%) and Msil_3609 (E-value, 420e-157; 62.24%), respectively (Figures 1b and c). Another open reading frame, Spo1579, found in an apparent operon with the other three open reading frames, shared homology with Msil_3605 (E-value, 2.0e-67; 35.75%), both of which contain a conserved tetrahydrofolate (H_4_F)-binding domain (GcvT). The GcvT domain is highly conserved in DmmD homologs (Zhu *et al.*, 2014) and is also found in bacterial TMAO demethylase (Tdm) (Lidbury *et al.*, 2014). Spo1579, Spo1580, Spo1581 and Spo1582 are hereafter referred to as *dmmD, dmmA, dmmB* and *dmmC*, respectively. Unlike in *M. silvestris*, *dmmD* was always co-located with *dmmABC* in the genomes of various MRC isolates screened (Supplementary Table S3), suggesting that its expression is tightly coordinated to that of *dmmABC.* Interestingly, *dmmDABC* was absent from the genome of *Candidatus* Pelagibacter ubique HTCC1062 (Figure 1d), a member of the SAR11 clade that can utilize TMA and MMA (Sun *et al.*, 2011).

### DmmABC forms a functional Dmm

**Figure 2:**
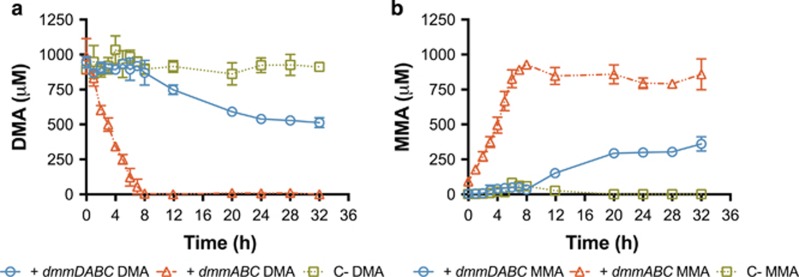
Assessment of (**a**) DMA degradation and (**b**) MMA accumulation in recombinant *E*. *coli* following heterologous expression of either the complete *dmmDABC* gene cluster from *R*. *pomeroyi* or just the structural genes (+ *dmmABC*), or of the expression vector pET28a as a negative control (C-). Results presented are the mean of triplicates and error bars denote s.d.

To determine if all four subunits of Dmm were essential for DMA demethylation, both *dmmDABC* and *dmmABC* were cloned into the expression vector pET28a and transformed into *E. coli* BLR(DE3)pLysS. In the *E. coli* strain harboring *dmmABC*, complete degradation of DMA (1 mM) occurred within 8 h while the concentration of MMA increased in a stoichiometric 1:1 manner (Figure 2). In the *E. coli* strain harboring *dmmDABC*, DMA degradation in accordance with MMA production still occurred, albeit at a slower rate, again, stoichiometrically in a 1:1 ratio (Figure 2). In cultures complemented with the empty pET28a vector, no DMA degradation and thus no MMA production was observed (Figure 2), whereas cultures grew comparably (Supplementary Figure S1). Together, these results show that the three-subunit cluster alone forms a functional Dmm.

### *dmmD* is essential for growth on DMA and other MAs in R. pomeroyi

**Figure 3:**
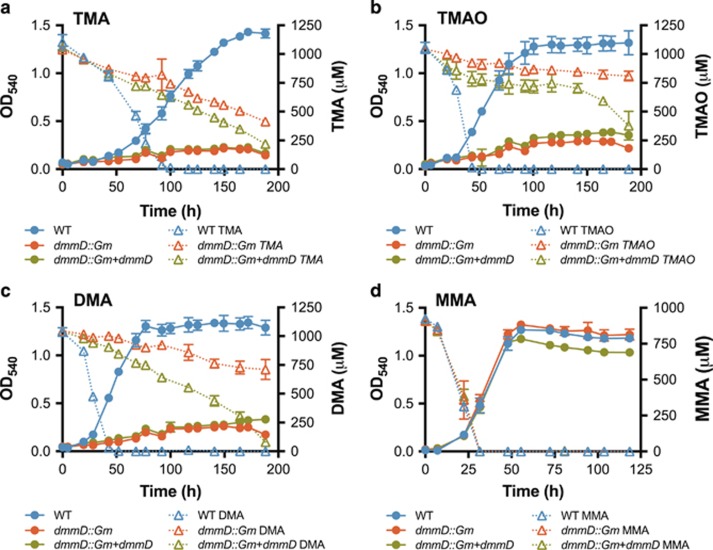
Growth of *R. pomeroyi* DSS-3 wild-type (WT), *dmmD* mutant (*dmmD*::Gm) and its complementation with *dmmD* (*dmmD::Gm*+*dmmD*) on (**a**) TMA, (**b**) TMAO, (**c**) DMA and (**d**) MMA as the sole nitrogen source. Solid lines represent cell growth. Dashed lines represent the degradation of the appropriate substrate with the concentrations of TMA, TMAO, DMA and MMA being quantified throughout the whole experiment. Results presented are the mean of triplicates and error bars denote s.d.

To determine the function of *dmmD* in *R. pomeroyi*, the gene was disrupted by insertion of a gentamicin resistance marker (Dennis and Zylstra, 1998) and the *dmmD::Gm* mutant subsequently grown on MAs including DMA as a sole nitrogen source. Disruption of the *dmmD* gene resulted in an inability of the mutant to grow on TMA, TMAO or DMA as a sole nitrogen source (Figures 3a–c). However, growth on MMA and NH_4_^+^ was unaffected (Figure 3d,Supplementary Figure S2a). Complementation with *dmmD* did not restore growth in comparison with the wild type (Figures 3a–c). *dmmDABC* forms a single operon and therefore deletion of *dmmD* may have affected the downstream expression of *dmmABC.* When grown on TMA and TMAO, the *dmmD* mutant accumulated DMA in the culture medium revealing a bottleneck in the MA degradation pathway (Supplementary Figure S3). However, when grown on DMA as the sole nitrogen source DMA degradation was slightly enhanced by complementation (Figure 3c), suggesting that *dmmD* may be required for DMA degradation in *R. pomeroyi*.

**Figure 4:**
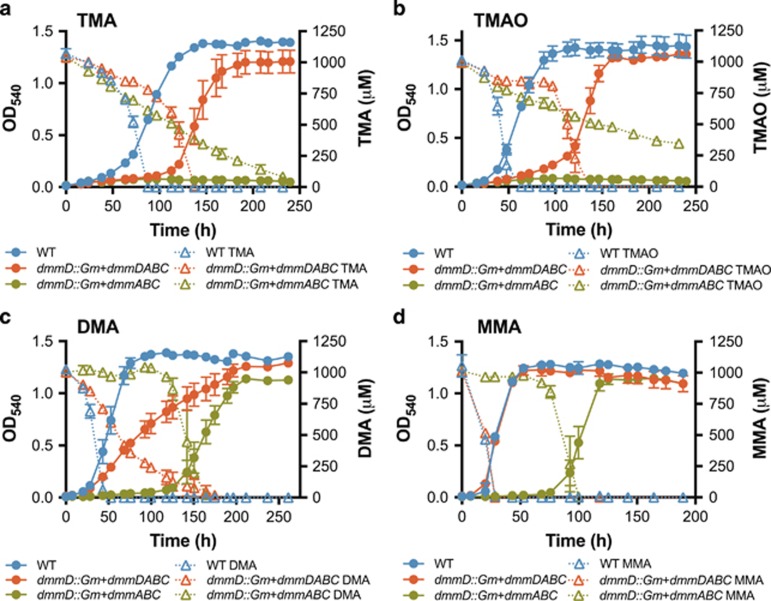
Growth of *R. pomeroyi* DSS-3 wild-type (WT), and the *dmmD* mutant (*dmmD::Gm*) complemented with either the four-gene cluster *dmmDABC* (*dmmD::Gm*+*dmmDABC*) or only the structural genes *dmmABC* (*dmmD::Gm*+*dmmABC*) along with the promoter on different nitrogen sources. Nitrogen was supplied in the form of (**a**) TMA, (**b**) TMAO, (**c**) DMA and (**d**) MMA. Solid lines represent cell growth. Dashed lines represent the degradation of the appropriate substrate with the concentrations of TMA, TMAO, DMA and MMA being quantified throughout the whole experiment. Results presented are the mean of triplicates and error bars denote s.d.

Owing to the potential polar effect on *dmmABC* by deletion of *dmmD*, the *dmmD* mutant was complemented with either the four-gene cluster *dmmDABC* (*dmmD::Gm*+*dmmDABC*) or the three subunits of Dmm, that is, *dmmABC* (*dmmD::Gm*+*dmmABC*). To achieve this, these two gene clusters were cloned into the broad-host range plasmid pBBR1MCS-km (Kovach *et al.*, 1995) together with the putative promoter located at the 5′ untranslated region upstream of *dmmD*. For the *dmmD::Gm*+*dmmDABC* complemented mutant, growth on TMA and TMAO as a sole nitrogen source was restored while for *dmmD::Gm*+*dmmABC*, missing an intact *dmmD*, the complemented mutant failed to grow on either TMA or TMAO (Figures 4a and b). Consequently, in the *dmmD::Gm*+*dmmABC* complemented mutant, DMA accumulated in the medium as TMA or TMAO degradation occurred (Supplementary Figure S4). However, both complemented strains could degrade and subsequently grow on DMA, MMA and NH_4_^+^ as sole nitrogen sources (Figures 4c and d, Supplementary Figure S5a), suggesting that *dmmD* is essential for TMA and TMAO degradation but not for growth on DMA or MMA in this bacterium.

### The distribution of DmmDABC in marine bacterial genomes and metagenomes

**Figure 5:**
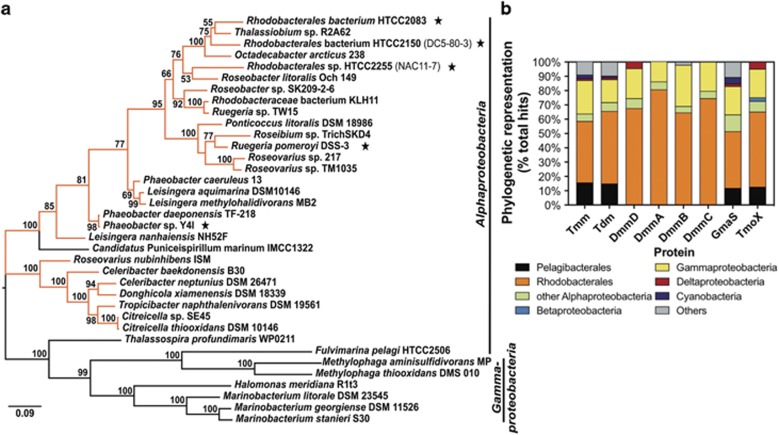
Distribution of genes for MA metabolism in marine bacterial isolates. (**a**) Maximum likelihood phylogenetic tree of *dmmDABC* homologs in marine bacterial isolates. For each node bootstrap values (999 replicates) >50% are given. MRC are marked in orange. An asterisk indicates pelagic *Roseobacter*, with the affiliation of two representatives to the largely uncultivated pelagic *Roseobacter* lineages according to Zhang *et al.* (2016) given in brackets. (**b**) Phylogenetic distribution of the genes encoding the enzymes involved in MA metabolism. TmoX, substrate-binding protein of the TMAO transporter, other abbreviations are as described in Figure 1.

The distribution of genes encoding DmmDABC was investigated using BLASTP analysis among marine bacterial genomes deposited in the IMG database of the JGI. In parallel, the distribution of genes encoding the other enzymes required for growth on MAs (for example, Tmm, Tdm, TmoX and GmaS) was also determined using *R. pomeroyi* homologs as the query sequences. The *dmmDABC* gene cluster was identified in 30 isolates related to *Alphaproteobacteria* and 6 related to *Gammaproteobacteria* (Figures 5a). The majority of *Alphaproteobacteria* homologs were related to the MRC (27/30). In addition, *dmmDABC* homologs were retrieved from *Candidatus* Puniceispirillum marinum IMCC1322 (IMCC1132), a member of the cosmopolitan SAR116 clade (Oh *et al.*, 2010; Giovannoni and Vergin, 2012) and clustered with the MRC homologs suggesting horizontal gene transfer has occurred (Figure 5a). A number of *dmmDABC* homologs were also found in the genomes of largely uncultivated pelagic *Roseobacter* (Figure 5a,Supplementary Table S3), some of which have been previously reported to possess features of a free-living life style (for example, *Rhodobacterales* sp. HTCC2255) (Billerbeck *et al.*, 2016; Zhang *et al.*, 2016). Notably, all representatives of the *Pelagibacterales* (SAR11 clade) lack homologs of the genes encoding DmmDABC (Figure 5, Supplementary Table S3), whereas genes encoding GmaS, Tmm, Tdm and TmoX were ubiquitous within the genomes of strains related to this clade (Figure 5b,Supplementary Table S3).

**Figure 6:**
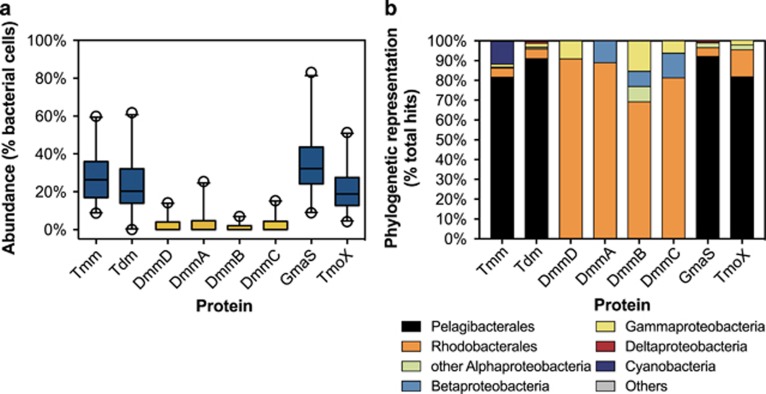
Distribution of genes encoding proteins for MA metabolism in selected marine metagenomes. (**a**) Abundance of selected genes in marine bacteria, and (**b**) their phylogenetic affiliation. In the box-whisker plot whiskers represent the 5 and 95 percentiles and the line corresponds to the median. Circles represent outliers with all high-range outliers of Tmm, Tdm, GmaS and TmoX deriving from the same Sargasso Sea metagenome sample (Supplementary Table S4). The phylogenetic composition represents the normalized relative abundances of MA-degrading genes using metagenomes primarily retrieved from the global ocean sampling (GOS) data set (Rusch *et al.*, 2007) (see Supplementary Table S4). Abbreviations are as described in Figure 1.

Previous studies have shown that *tmm*, *tdm* and *gmaS* are abundant in marine metagenomes primarily because of their occurrence in SAR11 clade bacteria (Chen *et al.*, 2011; Lidbury *et al.*, 2014). We hypothesized that the abundance of *dmmDABC* in marine metagenomes would be lower than that of *tmm, tdm* and *gmaS,* reflecting their absence from the genomes of SAR11 clade bacteria. To test this hypothesis, a number of metagenomes deposited in the IMG/JGI database, predominantly from the global ocean sampling expedition (Rusch *et al.*, 2007) were screened (stringency, e-50) for the presence of *dmmDABC* as well as *tmm, tdm, tmoX* and *gmaS* using the *R. pomeroyi* homologs as the query sequences. To determine the percentage of marine bacteria possessing MA degradation genes present at each site, counts were normalized against the average counts of two single copy genes (*recA* and *gyrB*). As expected *tmm*, *tdm, tmoX* and *gmaS* were present in 20–25% of marine bacteria (Figure 6a). However, *dmmDABC* was found at a much lower abundance (Figure 6a, Supplementary Table S4). To rule out the possibility that the under-representation of *dmmDABC* genes in marine metagenomes was because of the use of a high stringency cutoff value (e-50), we re-analyzed metagenomes from the global ocean sampling data set with a range of stringency thresholds (e-40, e-20, e-10, e-8) and the number of hits related to *dmmDABC* did not increase relative to that of *tmm* and *tdm* (Supplementary Figure S6). *dmmDABC* were also retrieved from metagenomes associated with high primary productivity, for example, a photosynthetic picoeukaryote bloom in the Norwegian Sea (IMG genome ID 3300002186), albeit at a lower abundance than other MA-degrading genes (Supplementary Table S4). Phylogenetic analysis revealed that *dmmDABC* sequences retrieved from marine metagenomes were primarily related to the MRC (Figure 6b). It should be noted that several *tmm* and *tdm* sequences were related to the newly identified gammaproteobacterium, *Candidatus* Thioglobus singularis (Marshall and Morris, 2015). A similar pattern was also observed when scrutinizing metatranscriptomes (Supplementary Table S5). No transcripts related to *dmmDABC* could be detected from various open ocean and coastal ocean waters, whereas transcripts related to various other genes involved in the MA degradation pathway (*tmm, tdm, gmaS* or *tmoX*) were readily detected (Supplementary Table S5, Ottesen *et al.*, 2011, 2013; Gifford *et al.*, 2013).

## Discussion

Recently, the genes involved in DMA degradation were identified in methylotrophic soil bacteria (Zhu *et al.*, 2014; Dziewit *et al.*, 2015). However, neither study conclusively demonstrated the functionality of Dmm at the protein level. By identifying *R. pomeroyi dmmDABC* homologs similar to those found in *M. silvestris* and *P. aminophilus* we were able to confirm that *dmmABC* does indeed encode a functional Dmm, an enzyme originally described in *A. aminovorans* (Alberta and Dawson, 1987). In both *M. silvestris* and *P. aminophilus*, *dmmD* was not essential for growth on MAs, but disruption of this gene did affect their growth rates on TMA, DMA and TMAO (the latter substrate was shown for *M. silvestris* only) (Zhu *et al.*, 2014; Dziewit *et al.*, 2015). These findings, alongside the data presented here (Figures 2–4), further suggest that *dmmD* is required for normal growth on MAs. As DmmD possesses a H_4_F-binding domain, its primary role is likely to be involved in the conjugation of free formaldehyde, released from the demethylation of DMA, with the one carbon (C1) carrier molecule H_4_F (Zhu *et al.*, 2014). Unlike *M. silvestris* and *P. aminophilus*, marine bacteria only possess the genes for C1 oxidation via the H_4_F pathway, lacking the genes required for C1 oxidation through either the tetrahydromethanopterin (H_4_MPT), glutathione-linked pathway or the formaldehyde-activating enzyme (Chistoserdova, 2011; Dziewit *et al.*, 2015). Thus, there is a greater dependency of the H_4_F-linked C1 oxidation pathway to deal with formaldehyde stress. The consistently tight genetic arrangement of *dmmDABC* in marine bacteria coupled with the non-essential function of *dmmD* in DMA or MMA degradation further strengthens the hypothesis that *dmmD* serves a key role in reducing formaldehyde toxicity. Furthermore, conjugation with H_4_F also allows the C1 unit to be fully oxidized to CO_2_ while generating reducing power (Lidbury *et al.*, 2015).

The absence of *dmmDABC* from members of the SAR11 clade as well as abundant marine *Gammaproteobacteria* and *Deltaproteobacteria* is intriguing. *C.* Pelagibacter ubique HTCC1062 has been shown to oxidize TMA, TMAO and MMA in order to generate adenosine triphosphate (Sun *et al.*, 2011). However, currently there is no evidence that this bacterium or any other member of the SAR11 clade can oxidize DMA. Furthermore, there is no evidence that SAR11 clade bacteria can grow on MAs as a source of nitrogen, which would require the complete demethylation of MAs, including DMA (Lidbury *et al.*, 2015). During N-limitation, *C.* Pelagibacter ubique HTCC1062 does express a protein that is predicted to be a general amine oxidase (Smith *et al.*, 2013), but its role in DMA oxidation has not been confirmed experimentally. In contrast to the *Pelagibacterales*, *dmmDABC* is found in pelagic *Roseobacters* (Figure 5a, Supplementary Table S3), thus, ruling out an affiliation of its absence with a pelagic life style. Representatives possessing the *dmm* genes have been found in the streamlined, largely non-cultivated pelagic *Roseobacter* lineages DC5-80-3 and NAC11-7 (Zhang *et al.*, 2016), whereas the other globally abundant pelagic *Roseobacter* CHAB-I-5 lineage (Billerbeck *et al.*, 2016; Zhang *et al.*, 2016) only shows genetic evidence for oxidation of TMA, TMAO and MMA, but not DMA (that is, no *dmm* genes found in their genomes).

The flux of MAs from surface seawaters is important as these compounds can lead to the formation of aerosols and thus cloud condensation nuclei (Ge *et al.*, 2011). Owing to the scarcity of labile organic nitrogen in marine surface waters, biological consumption of MAs as a nitrogen source is likely to be a major limitation on the air–sea exchange of these compounds (Balch, 1985; Carpenter *et al.*, 2012; Chen, 2012). In addition, *R. pomeroyi* and *C.* Pelagibacter ubique rapidly turn over MAs as an energy source (Sun *et al.*, 2011; Lidbury *et al.*, 2015), further reducing the amount of MAs available for air–sea exchange. The lack of *dmmDABC* homologs relative to other MA degradation genes (*tmm, tdm*and *gmaS*) in marine metagenomes suggests that DMA may accumulate in surface waters and therefore be susceptible to a greater amount of air–sea exchange. In support of this hypothesis, besides methanesulfonic acid, DMA amine salts were often the most abundant SOAs detected in fine marine particles at sites located in the North and Tropical Atlantic Ocean (Facchini *et al.*, 2008; Müller *et al.*, 2009). In these studies, a link between elevated concentrations of amine-derived SOAs detected in fine marine particles and elevated levels of primary production was observed and thought to be of biological origin. In another study, a shift toward amine-derived SOAs and the subsequent accumulation of cloud condensation nuclei was correlated with elevated periods of primary production (Sorooshian *et al.*, 2009). In this context, metagenomic data collected during a photosynthetic picoeukaryote bloom in the Norwegian Sea revealed that *dmmDABC* homologs were substantially reduced (5.95% of total bacteria) compared with those of *tmm, tdm* and *gmaS* (42.83% of total bacteria) (Supplementary Table S4). Similarly, in the North Sea where members of the MRC are often numerically abundant during phytoplankton blooms (Teeling *et al.*, 2012; Wemheuer *et al.*, 2015), *dmmDABC* homologs were again under-represented (6% of total bacteria) relative to other MA degradation genes (21% of total bacteria) (an average of 41 metagenomes, Supplementary Table S4). Therefore, a lack of DMA-degrading bacteria relative to other MA-degrading bacteria in the euphotic zone, especially during periods of elevated primary production, may be an explanation for the higher abundance of DMA-containing SOAs.

In conclusion, this study has confirmed the genes and enzyme catalyzing DMA degradation in marine bacteria and revealed a potential bottleneck in the MA degradation pathway in surface seawaters. We propose that this metabolic bottleneck likely explains the elevated abundance of DMA-derived amine salts detected in fine marine particles. Further research on the environmental cycling of MAs, especially DMA, is required to better understand the air-sea exchange of these climatically important compounds.
