# Supplementary material for: Identification of dimethylamine monooxygenase in marine bacteria reveals a metabolic bottleneck in the methylated amine degradation pathway
Source: ISME J. 2017 Mar 17;11(7):1592–601. doi: 10.1038/ismej.2017.31 (PMC5520151; doi:10.1038/ismej.2017.31)
Supplement: Supplementary Figures [file ismej201731x1.pdf]

Supplementary Figures

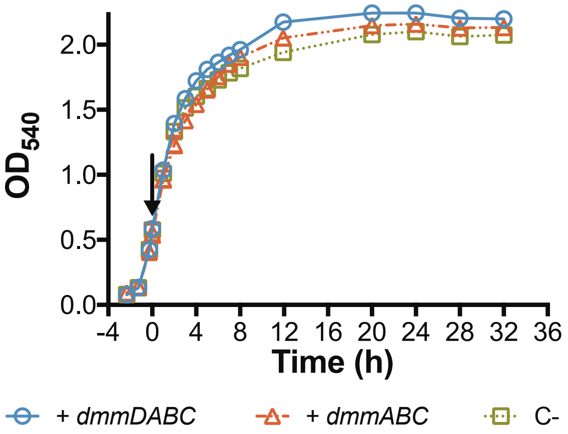

Supplementary Figure S1. Growth of transformed *E. coli* BLR(DE3)pLysS cells containing the *dmmDABC* genes, the structural genes *dmmABC*, or the expression vector pET28a as negative control (C-) in the presence of 1 mM DMA. The arrow indicates the time of IPTG addition (0.2 mM). Results presented are the mean of triplicates. Error bars denote standard deviation.

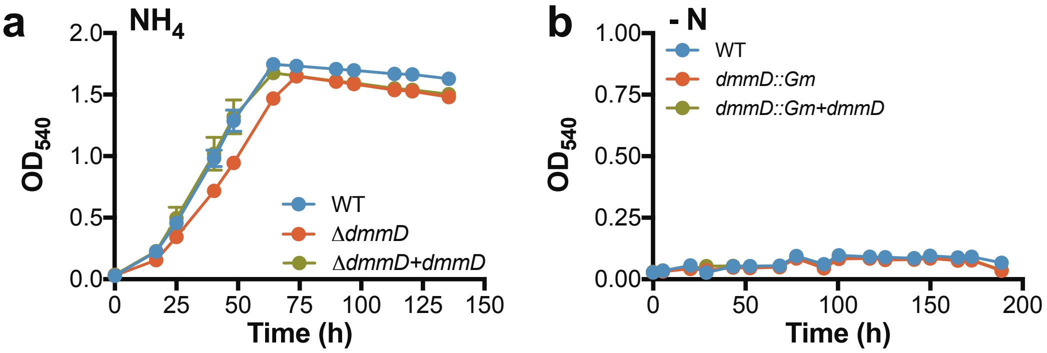

Supplementary Figure S2. Growth of *R. pomeroyi* DSS-3 wild-type (WT), *dmmD* mutant (*dmmD::Gm*), and its complementation with *dmmD* (*dmmD::Gm+dmmD*) on (**a**) ammonium as the sole nitrogen source or (**b**) without nitrogen source. Results presented are the mean of triplicates and error bars denote standard deviation.

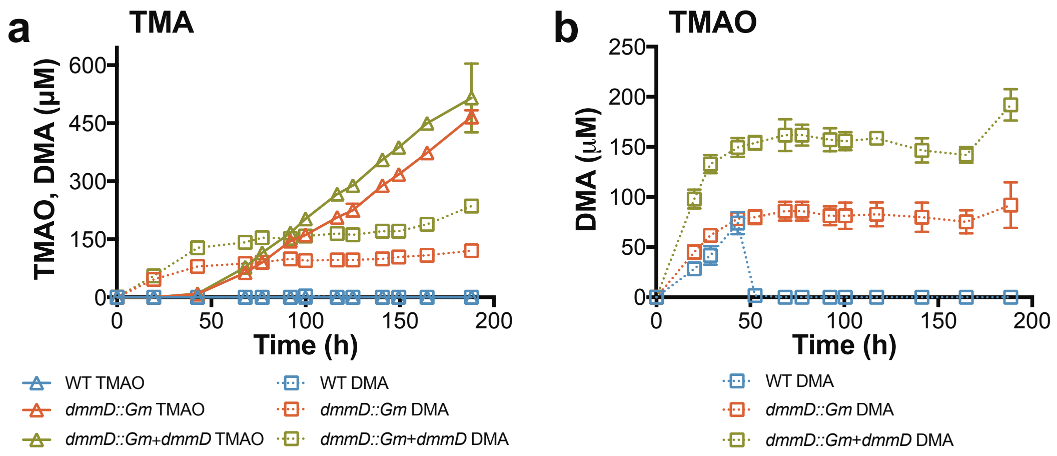

Supplementary Figure S3. Quantification of host and degradation products formed during the growth of *R. pomeroyi* DSS-3 wild-type (WT), *dmmD* disrupted mutant (*dmmD::Gm*), and its complementation with *dmmD* (*dmmD::Gm+dmmD*) on (**a**) TMA, and (**b**) TMAO. Results presented are the mean of triplicates and error bars denote standard deviation.

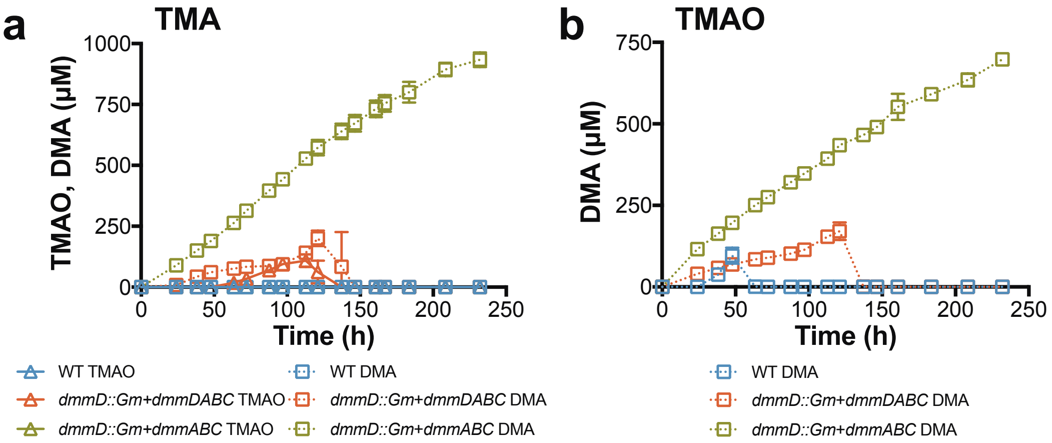

Supplementary Figure S4. Quantification of host and degradation products formed during growth of *R. pomeroyi* DSS-3 wild-type (WT), and the *dmmD* mutant (*dmmD::Gm*) complemented with the four-gene cluster *dmmDABC* (*dmmD::Gm+dmmDABC*) or the structural genes *dmmABC* (*dmmD::Gm+dmmABC*) plus promoter on (**a**) TMA, and (**b**) TMAO. Results presented are the mean of triplicates. Error bars denote standard deviation.

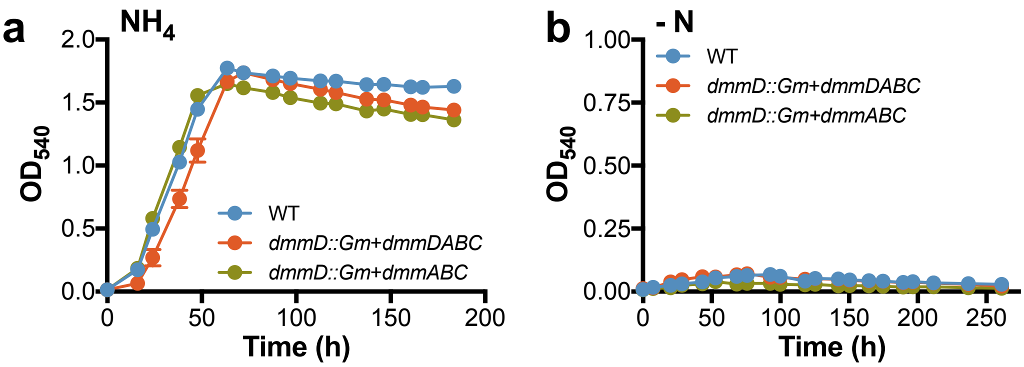

Supplementary Figure S5. Growth of *R. pomeroyi* DSS-3 wild-type (WT), and the *dmmD* mutant (*dmmD::Gm*) complemented with the four-gene cluster *dmmDABC* (*dmmD::Gm+dmmDABC*) or the structural genes *dmmABC* (*dmmD::Gm+dmmABC*) plus promoter on (**a**) ammonium as sole nitrogen source or (**b**) without nitrogen. Results presented are the mean of triplicates. Error bars denote standard deviation.

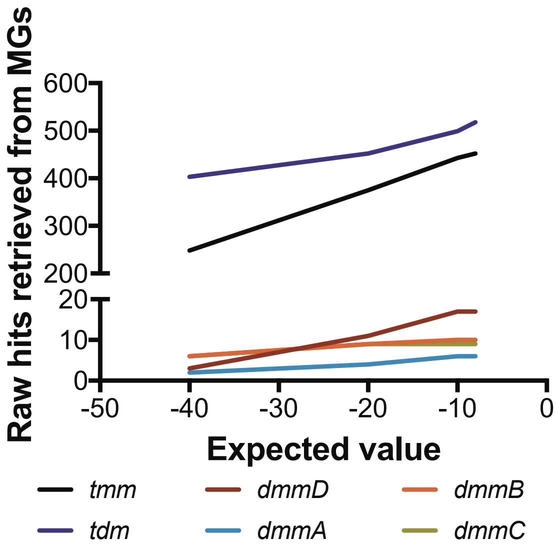

Supplementary Figure S6. Effect of stringency level on the retrieval of gene sequences from three large GOS metagenomes. Stringency thresholds applied were e^{-40}, e^{-20}, e^{-10}, and e^{-8}. Abbreviations as in Figure 1.
