# Supplementary material for: Identification of dimethylamine monooxygenase in marine bacteria reveals a metabolic bottleneck in the methylated amine degradation pathway
Source: ISME J. 2017 Mar 17;11(7):1592–601. doi: 10.1038/ismej.2017.31 (PMC5520151; doi:10.1038/ismej.2017.31)
Supplement: Supplementary Information [file ismej201731x2.docx]

**Supplementary Table Legends**

**Supplementary Table S1.** Bacterial strains and plasmids used in this study.

**Supplementary Table S2.** PCR primers used in this study.

**Supplementary Table S3.** Distribution of genes for methylated amine (MA) metabolism in marine bacterial isolates. A shaded box indicates the co-location of *dmmD* with *dmmABC* in MRC isolates’ genomes and a pale blue background indicates pelagic *Roseobacter* isolates. D, draft; F, finished; P, permanent; other abbreviation as described in Figure 1.

**Supplementary Table S4.** List of marine metagenomes and detailed breakdown of the abundance of each gene encoding the enzymes involved in the MA degradation pathway detected in the metagenomes retrieved at specific study sites. Asterisks indicate data normalised against RecA protein length. RecA, recombinase A; other abbreviations as in Figure 1.

**Supplementary Table S5.** Transcriptional abundance of genes involved in MA catabolism retrieved from various metatranscriptomes deposited in the IMG/JGI database. Asterisks indicate transcripts normalised against RecA protein length as described in the text. RecA, recombinase A; other abbreviations as in Figure 1.
